# Supplementary material for: OrthoID: profiling dynamic proteomes through time and space using mutually orthogonal chemical tools
Source: Nat Commun. 2024 Feb 29;15:1851. doi: 10.1038/s41467-024-46034-z (PMC10904832; doi:10.1038/s41467-024-46034-z)

Supplementary Information

OrthoID: Profiling Dynamic Proteomes Through Time and Space Using Mutually Orthogonal Chemical Tools

Ara Lee,[1,2,+] Gihyun Sung,[1,2,+] Sanghee Shin,[3,4] Song-Yi Lee,[5] Jaehwan Sim,[1,6] Truong Thi My Nhung,[7] Tran Diem Nghi,[7] Sang Ki Park,[7] Ponnusamy Pon Sathieshkumar,[1] Imkyeung Kang,[8,9] Ji Young Mun,[8] Jong-Seo Kim,*[3,4] Hyun-Woo Rhee,*[5] Kyeng Min Park,*[10] and Kimoon Kim*[1,2,6,11]

[1]Center for Self–assembly and Complexity, Institute for Basic Science (IBS), Pohang, 37673, Republic of Korea, [2]Division of Advanced Materials Science (AMS), Pohang University of Science and Technology (POSTECH), Pohang, 37673, Republic of Korea, [3]Center for RNA Research, Institute for Basic Science (IBS), Seoul 08826, Republic of Korea, [4]School of Biological Sciences, Seoul National University, Seoul 08826, Republic of Korea, [5]Department of Chemistry, Seoul National University, Seoul 08826, Republic of Korea, [6]School of Interdisciplinary Bioscience and Bioengineering, Pohang University of Science and Technology (POSTECH), Pohang 37673, Republic of Korea, [7]Department of Life Sciences, Pohang University of Science and Technology (POSTECH), Pohang, Republic of Korea, [8]Neural circuit research group, Korea Brain Research Institute, Daegu 41062, Republic of Korea, [9]Department of Microbiology, University of Ulsan College of Medicine, Ulsan 44601, Republic of Korea, [10]Department of Biochemistry, Daegu Catholic University School of Medicine, Daegu 42471, Republic of Korea, [11]Department of Chemistry, Pohang University of Science and Technology (POSTECH), Pohang 37673, Republic of Korea.
[+]These authors contributed equally to this work.

*Corresponding authors:
J.-S.K (jongseokim@snu.ac.kr),
H.-W.R (rheehw@snu.ac.kr),
K.M.P (kpark@cu.ac.kr),
K.K (kkim@postech.ac.kr)

Table of Contents

Supplemental tables and figures

Supplementary Table 1. Construct information 4

Supplementary Figure 1. Schematic description for detailed process of OrthoID. 5

Supplementary Figure 2. Preparation and characterization of model proteins. 6

Supplementary Figure 3. SPOT-SupraID and SPOT-BioID using model protein mixture (AdBt-BSA, Ad-OVA, Bt-MYB) 7

Supplementary Figure 4. CLSM images with neither Ad- nor Bt-treated stable cells. 8

Supplementary Figure 5. Protein band shift assay of IP_3R protein. 9

Supplementary Figure 6. Data correlation between the triplicate results of the mass analysis in NT cells. 10

Supplementary Table 2. Detection methods and functions of known MAM proteins identified by OrthoID 11

Supplementary Figure 7. Characterization for dual-labeling of identified proteins from OrthoID. 12

Supplementary Figure 8. 3D-rendering images reconstructed from z-stacked CLSM images of identified proteins from OrthoID. 13

Supplementary Figure 9. Organelle fractionation of identified proteins from OrthoID. 14

Supplementary Table 3. Known functions of soluble ER proteins identified as MAM proteins by our system 15

Supplementary Figure 10. Labeled peptide sequence and topology of LRC59. 16

Supplementary Figure 11. Confirmation of LRC59 protein knockdown by siRNA and expression of siRNA-resistant LRC59-Myc-DDK protein. 17

Supplementary Figure 12. Effect of LRC59 in the formation of ER-mito contact site 18

Supplementary Figure 13. Data correlation between the triplicate results of the mass analysis in CCCP-treated cells 19

Supplementary Figure 14. STRING analysis of exclusively identified MAM proteins in CCCP-treated cells. 20

Supplementary Figure 15. Schematic description of roles of nine exclusively identified proteins from OrthoID in CCCP-treated cells at ER-mito junction. 21

Supplementary Table 4. Known functions of exclusively identified proteins from OrthoID in CCCP-treated

cells. 22

Supplementary Figure 16. Increased interactions between proteins at ERM, OMM and IMM detected by proximity ligation assay (PLA) under CLSM. 23

Supplemental References 24

Uncropped Scans of Blots in Supplementary Figures 27

Other Supplemental Materials for this manuscript include the following:
Supplementary Data 1 to 5 (Excel files of MS results)

Supplementary Table 1. Construct information

Name	Features	Promotor/Vector	Details
TOM20-V5-TurboID	*KpnI*-TOM20-*BamHI*-V5-*NheI*-BirA(humanized)-*XhoI*	CMV/pcDNA5	TOM20 (NM_014765.2)
Sec61B-V5-APEX2	*NotI*-Sec61B-*NheI*-V5-APEX2-Stop-*XbaI*	CMV/pcDNA3	Sec61B (NM_006808)
BFP-KDEL	*EcoRV*-ss-*ApaI*-*BglII*-EBFP-KDEL-Stop-*NotI*	CMV/pDisplay	ss: METDTLLLWVLLLWVPGSTGD (IgK chain signal sequence for ER lumen)<br>KDEL: ER retention motif
Mito-BFP	*XhoI*-Mito-*BamHI*-BFP-Stop-*NotI*	CMV/pC1	Mito: MSVLTPLLLRGLTGSARRLPVPRAK (COX8 sequence)
mScarlet-Sec61B-C1	*NheI*-mScarlet-*XhoI*-Sec61b-Stop-*EcoRI*	CMV/pC1 (pmScarlet-H_C1)	pmScarlet-H_C1 is from Addgene #85043<br>Sec61B (NM_006808.3)
siRNA-resistant LRC59 (LRC59-BFP)	*NheI*-LRC59-*KpnI*-BFP-Stop-*XbaI*	CMV/pcDNA3.1+	LRC59 (NM_018509)
LRC59-mEmerald	*NheI*-LRC59-*KpnI*-mEmerald-Stop-*XbaI*	CMV/pcDNA3.1+	A 15X linker (GGS)5 was added in between LRC59 and mEmerald.<br>LRC59 (NM_018509)
MESD-mEmerald	*NheI*-MESD-*KpnI*-mEmerald-Stop-*XbaI*	CMV/pcDNA3.1+	A 15X linker (GGS)5 was added in between MESD and mEmerald.<br>MESD (NM_015154)
KPYM-mEmerald	*NheI*-KPYM-*KpnI*-mEmerald-Stop-*XbaI*	CMV/pcDNA3.1+	A 15X linker (GGS)5 was added in between KPYM and mEmerald.<br>KPYM (NM_182470)
GANAB-mEmerald	*NheI*-GANAB-KpnI-mEmerald-Stop-*XbaI*	CMV/pcDNA3.1+	A 15X linker (GGS)5 was added in between GANAB and mEmerald.<br>GANAB (NM_198334)
pLL3.7-EGFP-LRC59 shRNA	*PstI*-mU6-LRC59 shRNA-*XhoI*	mU6 for shRNA, CMV for EGFP/pLL3.7-EGFP	shRNA targeting 3'UTR of LRC59 was used for knockdown.<br>(target sequence: GCTCAGTAAATCCAGTCTAAA)

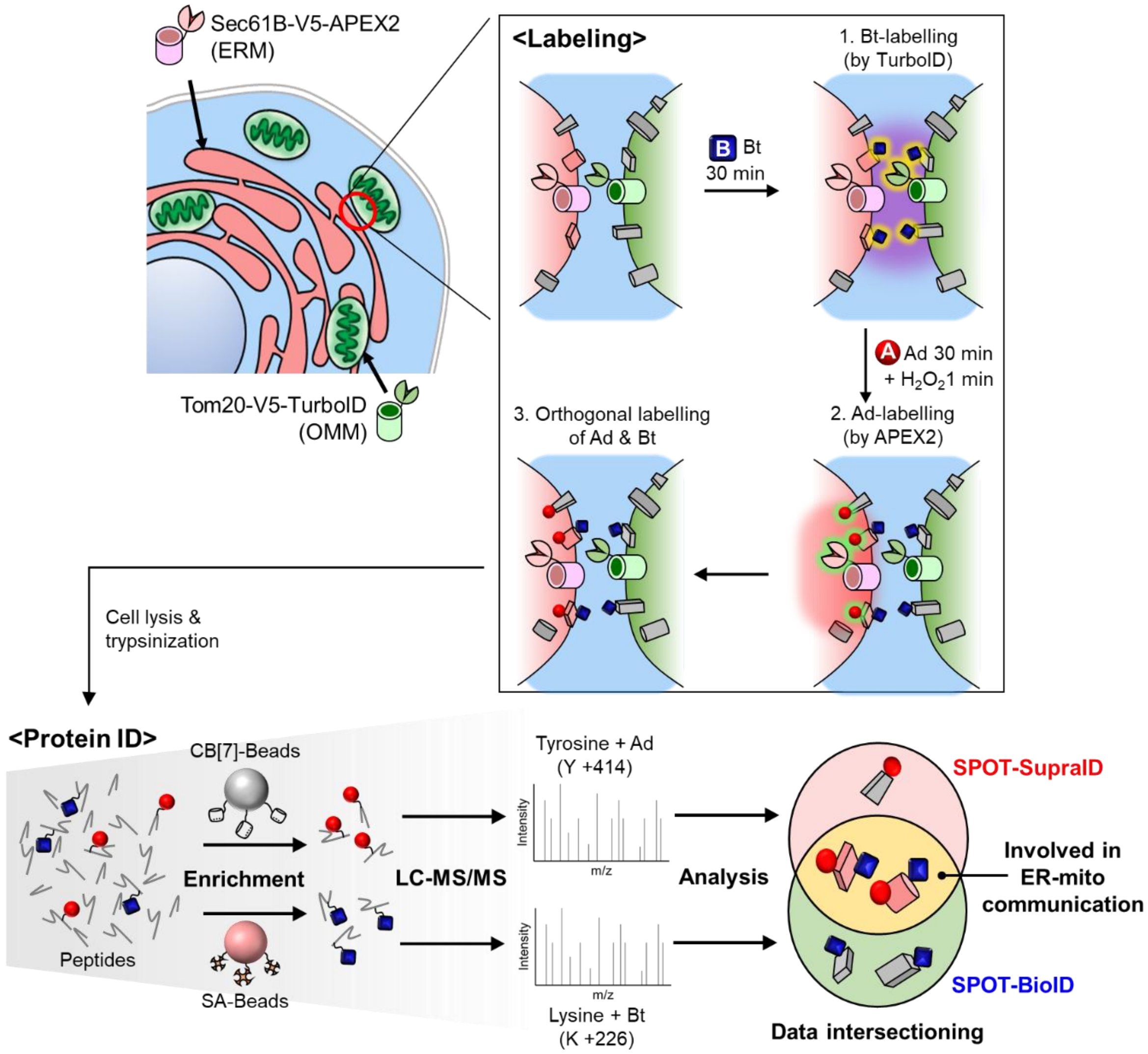

Supplementary Figure 1. Schematic description for detailed process of OrthoID.

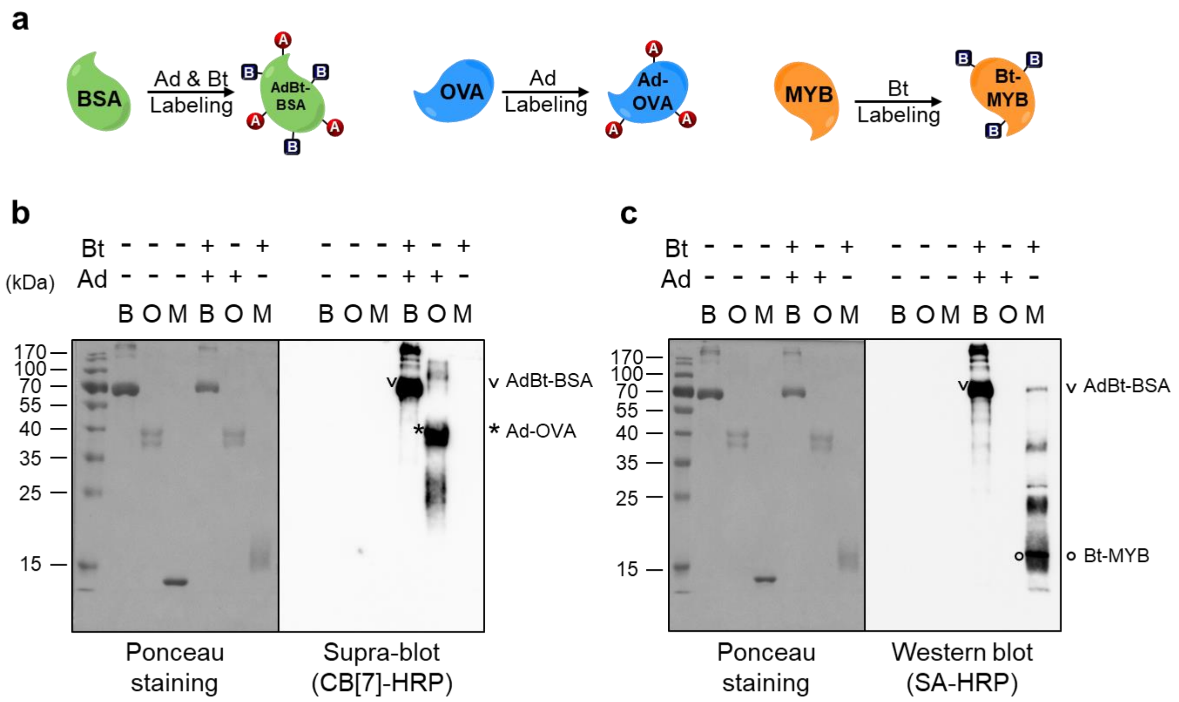

Supplementary Figure 2. Preparation and characterization of model proteins.
(a) Schematic description of Ad- or Bt-labeled BSA, OVA, and MYB.
(b) Supra-blot and (c) western blot using CB[7]-HRP and SA-HRP respectively. BSA (B) was characterized to be labeled with both Ad and Bt. OVA (O) and MYB (M) were characterized to be labeled with Ad and Bt, respectively. Data are representative of three independent experiments with similar results. Source data are provided as a Source Data file.

a

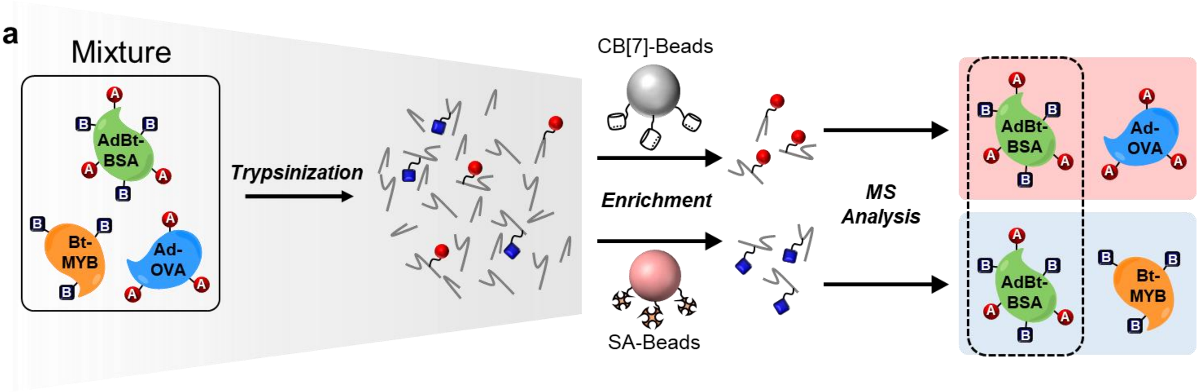

b

Analysis	SPOT-SupraID	SPOT-BioID
**Labeling**	Ad	Bt
**Total PSM**	1102	1879
**Labeled PSM**	827	1689
**BSA/OVA/MYB among labeled PSM**	256/544/0	356/0/1093

Supplementary Figure 3. SPOT-SupraID and SPOT-BioID using model protein mixture (AdBt-BSA, Ad-OVA, Bt-MYB)
(a) Schematic description for identification of model proteins by OrthoID using CB[7]- and SA-beads.
(b) Results of SPOT-SupraID and SPOT-BioID.

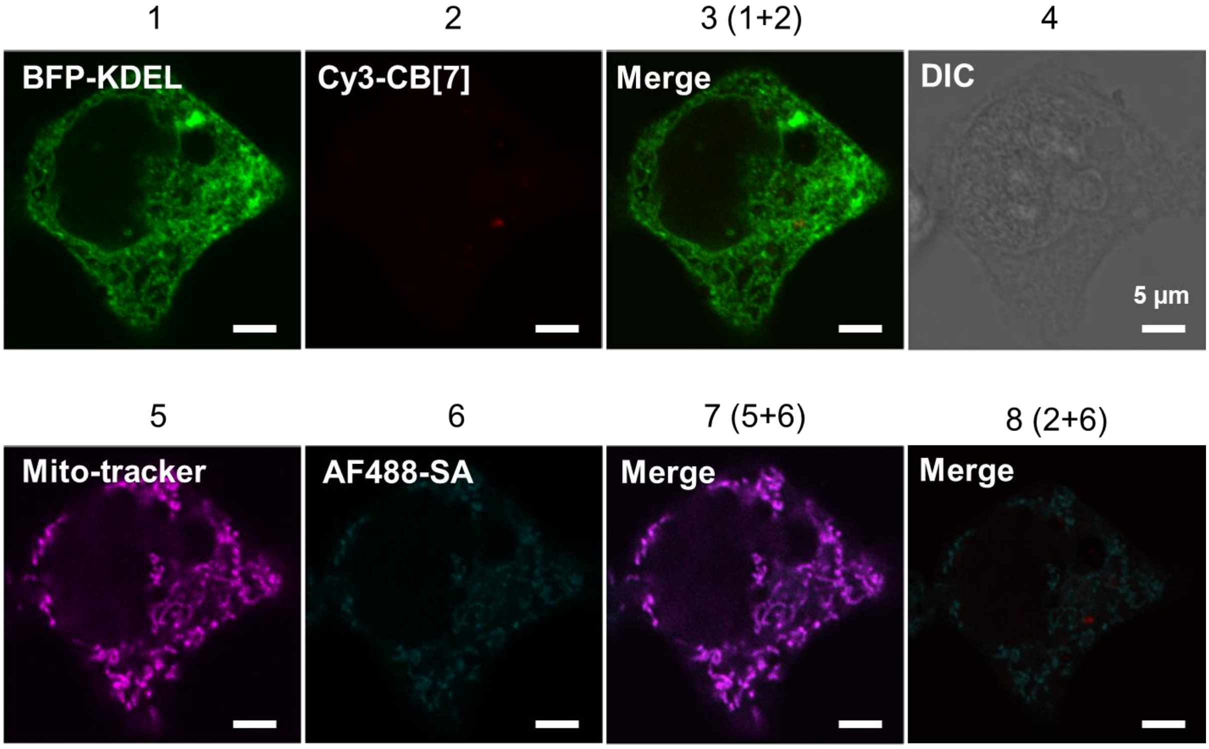

Supplementary Figure 4. CLSM images with neither Ad- nor Bt-treated stable cells. BFP-KDEL (green) as an ER-marker, Mito-tracker (purple) as a Mito-marker, Cy3-CB[7] (red) for Ad-labeled proteins, and AF488-SA (cyan) for Bt-labeled proteins. Data are representative of three independent experiments with similar results.

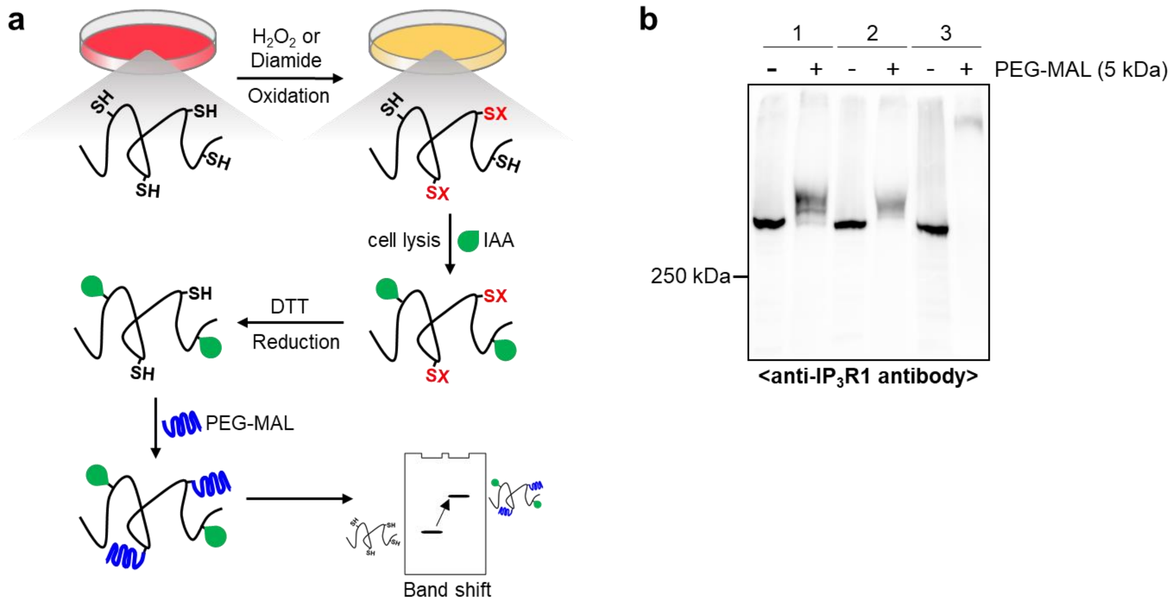

Supplementary Figure 5. Protein band shift assay of IP_3R1 protein.
(a) Schematic description of protein shift assay of IP_3R1. IAA represents iodoacetamide and PEG-MAL represents methoxypolyethylene glycol-maleimide (MW: 5k).
(b) Western blot using anti-IP_3R1 antibody. 1: non-treated, 2: H_2O_2 treated (1 mM, 1 min), 3: diamide treated (4 mM, 30 min). Data are representative of two independent experiments with similar results. Source data are provided as a Source Data file.

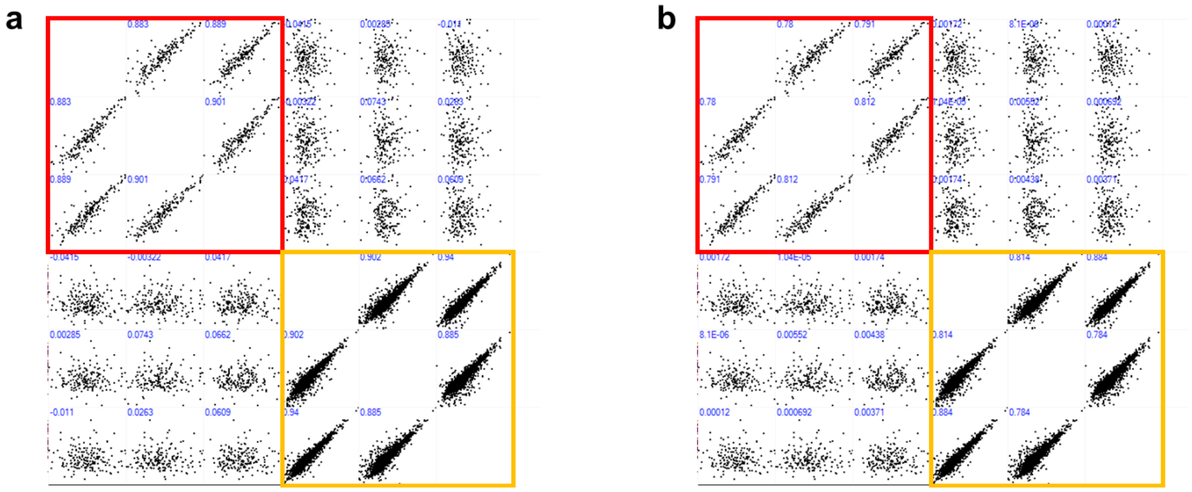

Supplementary Figure 6. Data correlation between the triplicate results of the mass analysis in NT cells.
(a) Pearson correlation (R) and (b) determination of coefficient (R^2) between triplicate datasets from SPOT-SupraID (red box, $R^2 > 0.78$) and SPOT-BioID (orange box, $R^2 > 0.78$) in NT cells.

Supplementary Table 2. Detection methods and functions of known MAM proteins identified by OrthoID
***References in research article (proximity labeling-based method)**
A: APEX coupled with biochemical fractionation[1], **B**: APEX2 at the ERM and OMM towards the cytosol[2]
C: Contact-ID (split pBirA)[3], **D**: Split-TurboID[4]
****References in review article**: **E**[5], **F**[6], **G**[7], **H**[8], **I**[9], **J**[10]

**Accession**	**Gene Name**	***References in research article (PL-based method)**	****References in review article**	**Confirmation method**	**MAM-related function**
Q96S66	CLCC1	A, C, D	J	Fluorescence microscopy[11]	Interacting partner of mitochondria microprotein (PIGBOS) to regulate unfolded protein response
Q9UGP8	SEC63	A, C			
P60468	SC61B	A, C			*ERM Translocon conjugated to APEX2
O94905	ERLN2		J	Organelle fractionation[12-13]	Cholesterol homeostasis, initiation of autophagy[14]
P13667	PDIA4	A			
P30101	PDIA3		G	Organelle fractionation[15]	Regulation of calcium homeostasis[16-17]
Q14739	LBR	C, D			
Q07065	CKAP4	C	J	Immunoprecipitation[18]	Regulation of mitochondria function, calcium influx
P50402	EMD	C, D	F	Organelle fractionation[19]	Spacer/linker at MAM
Q9NPA0	EMC7	C	F		Phospholipid transfer from ER to mitochondria[20]
O00429	DNM1L	D	E, F, G, I, J	Fluorescence microscopy[21-23]	Mitochondria fission
Q96AG4	LRC59	A, C		Fluorescence microscopy, Organelle fractionation (in this study)	Involved in the formation of ER-mito contact (in this study)
P51572	BAP31	A, D	F, H	Fluorescence microscopy[24]	Apoptosis signaling
Q8N5K1	CISD2	A, C			Maintenance of calcium homeostasis[25]
P21796	VDAC1	B	E, F, H ,I, J	Organelle fractionation[26]	Calcium signaling

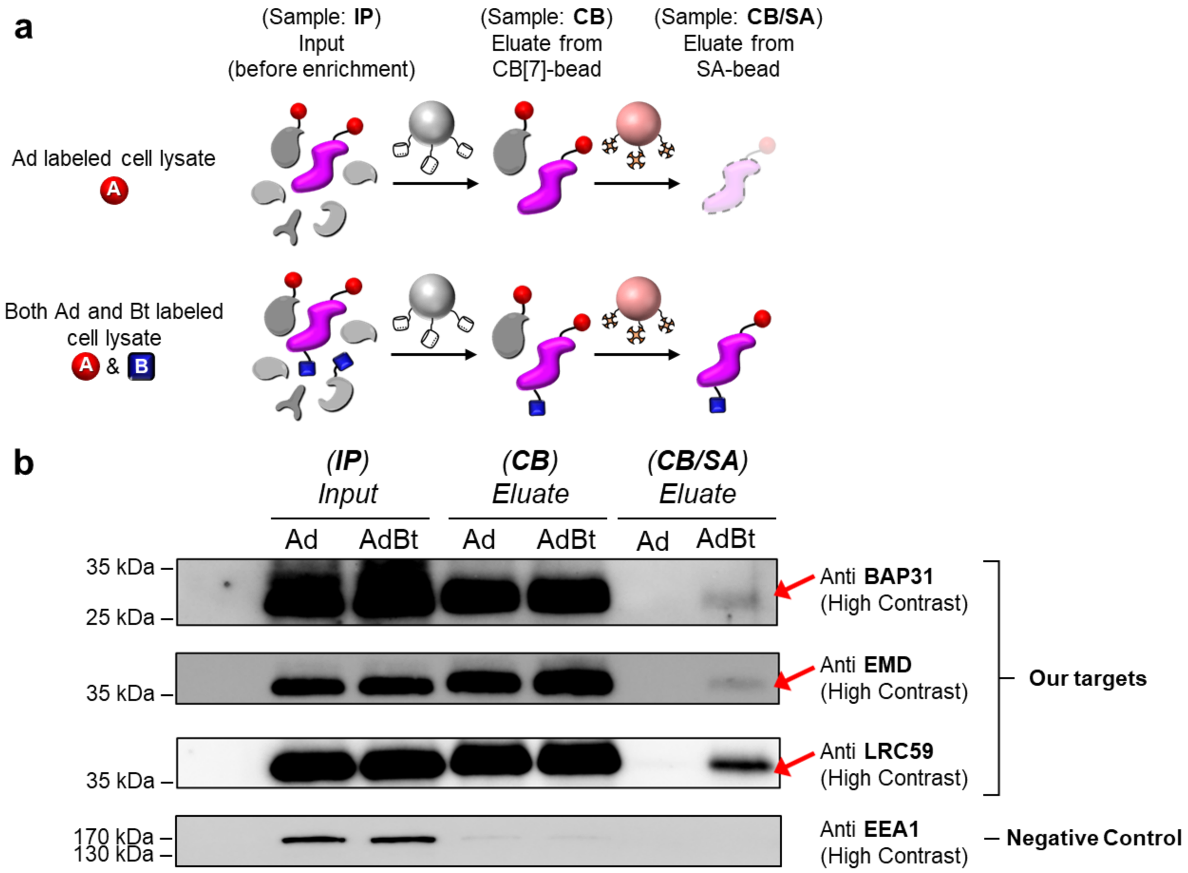

Supplementary Figure 7. Characterization for dual-labeling of identified proteins from OrthoID.
(a) Schematic description of protein-level sequential enrichment using CB[7]-beads followed by SA-beads.
(b) Western blotting of protein eluate from sequential enrichment with anti-BAP31 antibody, anti-EMD antibody and anti-LRC59 antibody. Anti-EEA1 antibody was tested as a negative control. Data are representative of two independent experiments with similar results. Source data are provided as a Source Data file.

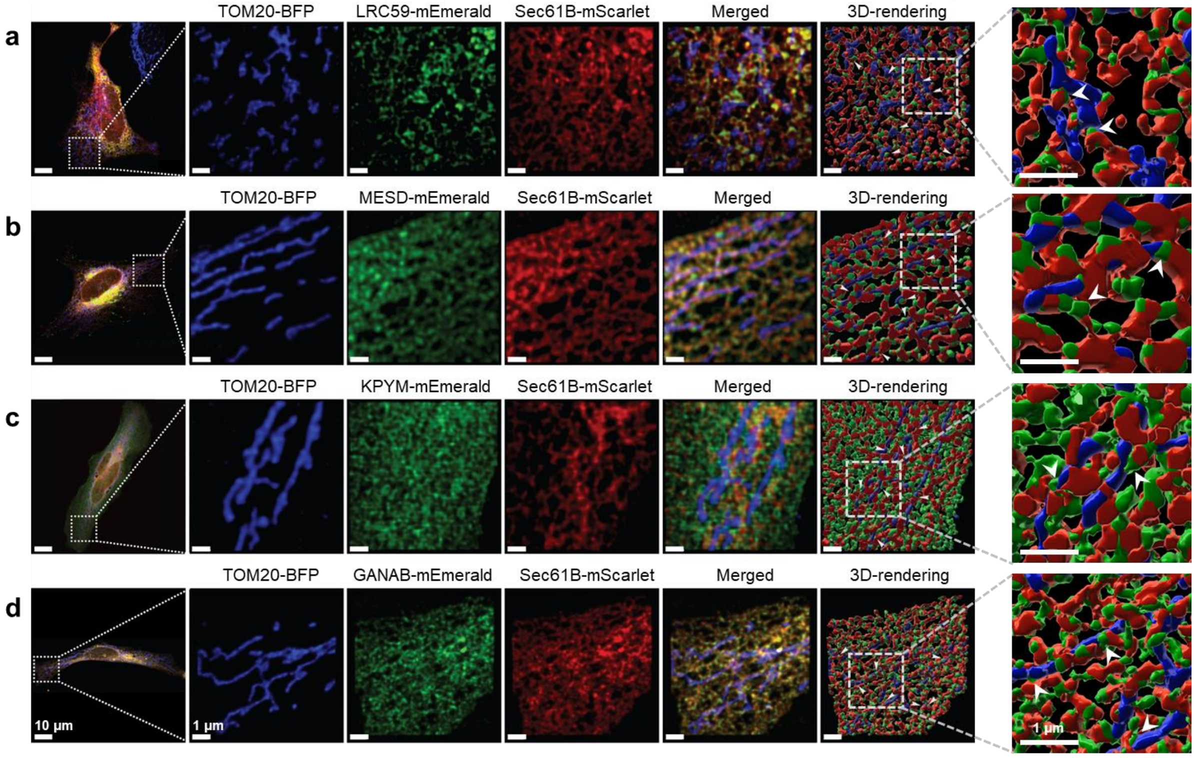

Supplementary Figure 8. 3D-rendering images reconstructed from z-stacked CLSM images of identified proteins from OrthoID.

(a) LRC59, (b) MESD, (c) KPYM, (d) GANAB fused to mEmerald (green) was visualized. TOM20-BFP (cyan) as outer mitochondrial membrane (OMM) marker, Sec61b-mScarlet (red) as ER marker. Scale bar: 10 µm, enlargement: 1 µm. Data are representative of three independent experiments with similar results.

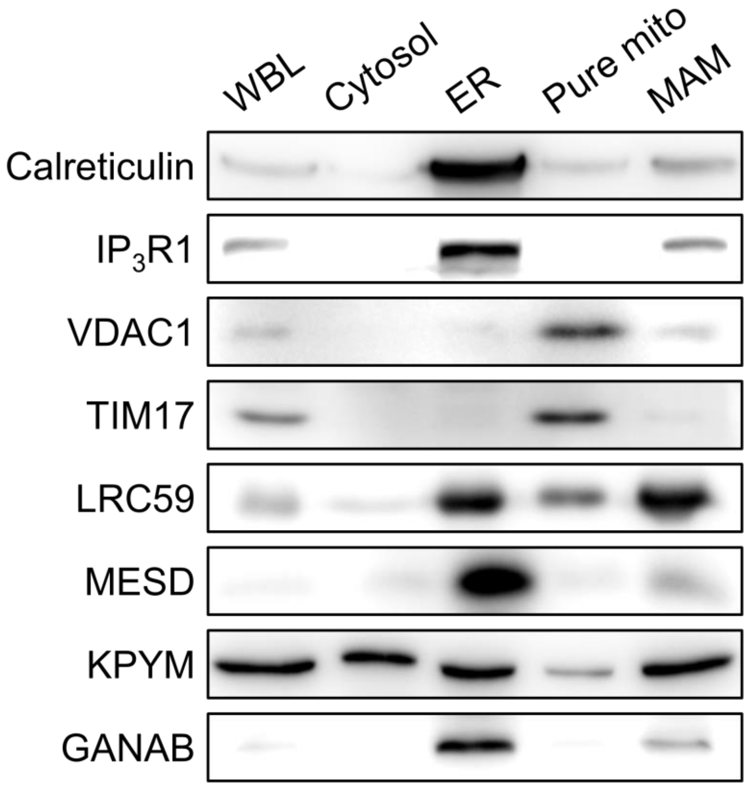

Supplementary Figure 9. Organelle fractionation of identified proteins from OrthoID. WBL: whole brain tissue lysate, ER: endoplasmic reticulum, Pure mito: pure mitochondria, MAM: mitochondria-associated ER membrane. Calreticulin and IP_3R1 were used as ER and MAM markers, VDAC1 as mitochondria and MAM markers, and TIM17 as pure mitochondria marker. Data are representative of three independent experiments with similar results. Source data are provided as a Source Data file.

Supplementary Table 3. Known functions of soluble ER proteins identified as MAM proteins by our system (*GOCC: Gene Ontology Cellular Component, PM: Plasma membrane)

Accession	Gene name	Localization (GOCC*)	Known function
Q14697	GANAB	ER	- Mannose trimming related to refolding of protein[27]<br>- Sigma1R protein interactome[28]
P14618	KPYM	ER	- Translation of ER-destined mRNA[29]<br>- Inhibition of apoptosis under oxidative stress[11]<br>- Interaction between methylated PKM2 and IP_3R result in downregulation of IP_3R[30]
Q14696	MESD	ER, PM*	- Chaperone for LRP5/6 protein (related to Wnt signaling, glucose uptake for mito regulation) folding & trafficking[31]

a

10 20 30 40 50
MTKAGSKGGN LRDKLDGNEL DLSLSDLNEV PVKELAALP**K** ATILDLSCNK
60 70 80 90 100
LTTLPSDFCG LTHLVKLDLS **K**NKLQQLPAD FGRLVNLQHL DLLNN**K**LVTL
110 120 130 140 150
PVSFAQL**K**NL **K**WLDLKDNPL DPVLAKVAGD CLDEKQCKQC ANKVLQHMKA
160 170 180 190 200
VQADQERERQ RRLEVEREAE KKREAKQRAK EAQERELRKR EKAEEKERRR
210 220 230 240 250
KEYDALKAAK REQEKKPKKE ANQAPKSKSG SRPRKPPPRK HTRSWAVLKL
260 270 280 290 300
LLLLLLFGVA GGLVACRVTE LQQQPLCTSV NTI**Y**DNAVQG LRRHEILQWV
LQTDSQQ

Cytosol
ER membrane
ER lumen

b

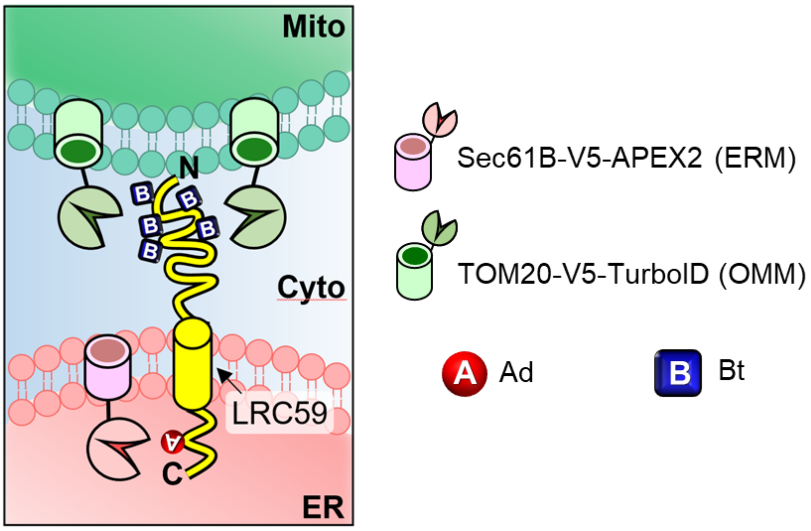

Supplementary Figure 10. Labeled peptide sequence and topology of LRC59.
(a) Known sequence of LRC59 from Uniprot database and Ad/Bt-labeled sites by OrthoID (K (lysine) with Bt (blue) and Y (tyrosine) with Ad (red)).
(b) Schematic description of labeling site-based topology of LRC59 by OrthoID.

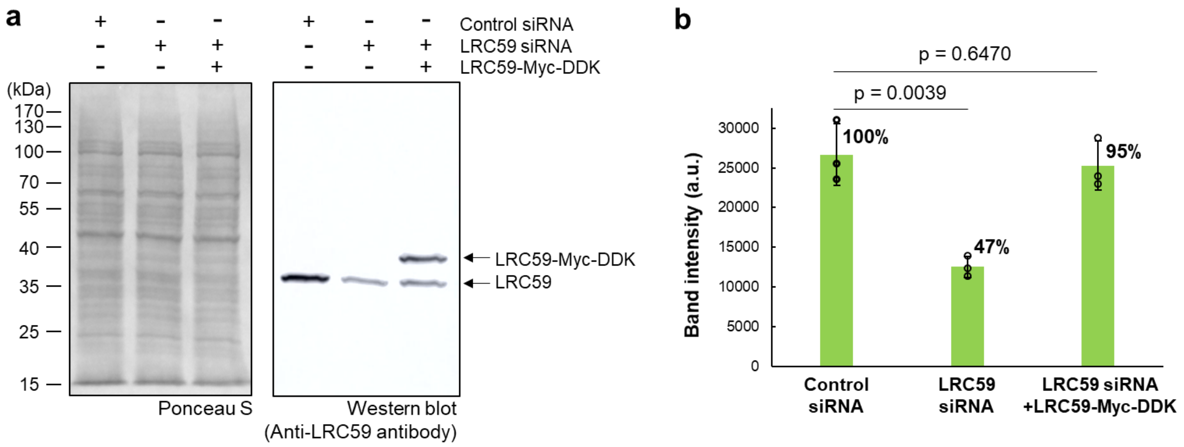

Supplementary Figure 11. Confirmation of LRC59 protein knockdown by siRNA and expression of siRNA-resistant LRC59-Myc-DDK protein.
(a) Western blot using anti-LRC59 antibody.
(b) Statistical analysis of western blot results. Error bars are the mean ± SD and the numbers in the graph represent exact p-values. (n = 3 independent experiments, two-tailed Student's t-test was used to assess statistical difference.) Source data are provided as a Source Data file.

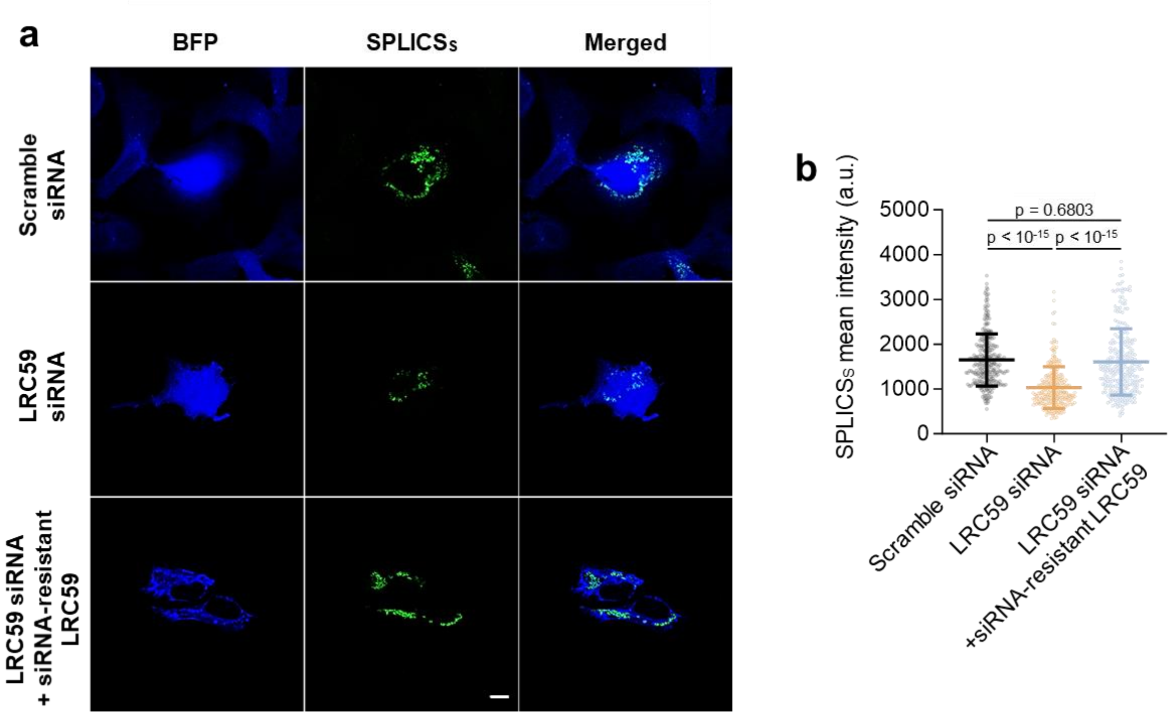

Supplementary Figure 12. Effect of LRC59 in the formation of ER-mito contact site

(a) CLSM images of a split green fluorescent protein-based contact site sensor (SPLICS) expressed cells in normal, LRC59 knockdown, and LRC59 re-expressed condition. ER-Short β_{11} was expressed in the ERM and OMM-GFP_{1-10} was expressed in the OMM. BFP was expressed as a cell marker. Scale bar: 10 µm.

(b) Quantitative analysis of the fluorescence intensity from SPLICSs (n = 279 cells for scramble siRNA, 262 for LRC59 siRNA cells and 252 cells for LRC59 siRNA+siRNA-resistant LRC59 examined in 3 independent experiments.) The error bar represents the mean ± SD and the numbers in the graph represent p-values. One-way ANOVA and Tukey's post hoc test for multiple comparisons were used to assess statistical differences. Source data are provided as a Source Data file.

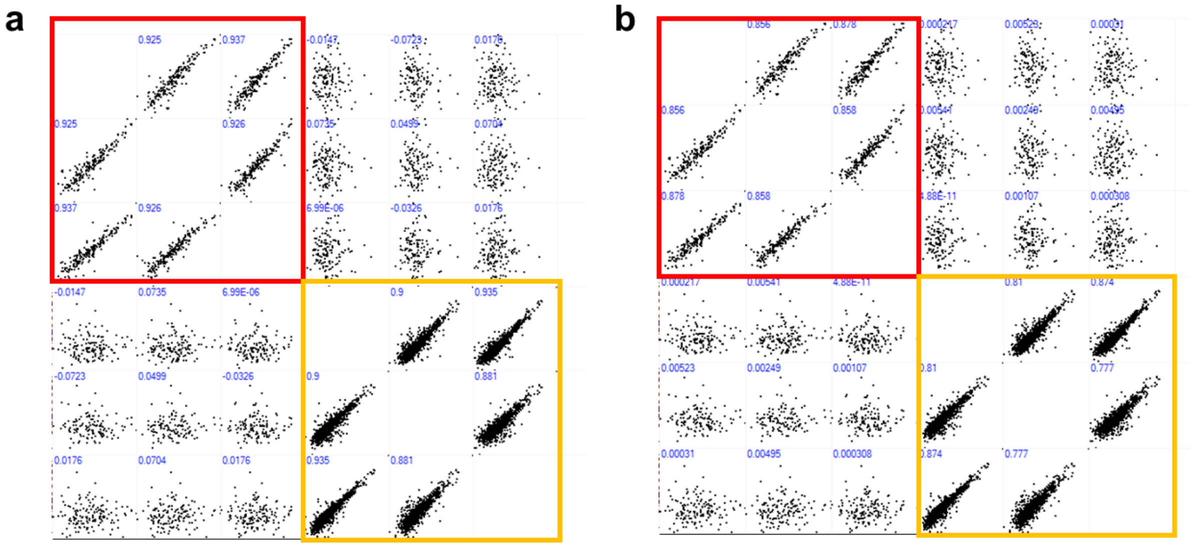

Supplementary Figure 13. Data correlation between the triplicate results of the mass analysis in CCCP-treated cells.

(a) Pearson correlation (R) and (b) determination of coefficient (R2) between triplicate datasets from SPOT-SupraID (red box, $R^2 > 0.86$) and SPOT-BioID (orange box, $R^2 > 0.78$) in CCCP-treated cells.

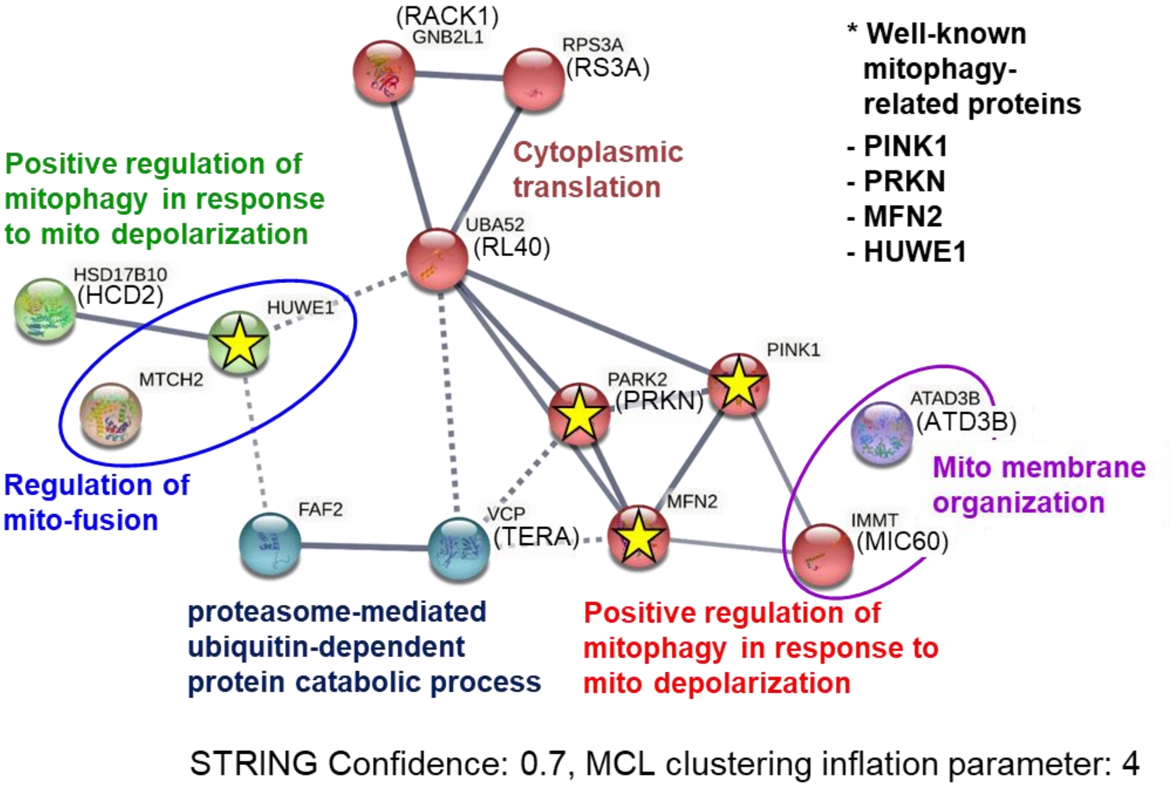

Supplementary Figure 14. STRING analysis of exclusively identified MAM proteins in CCCP-treated cells. STRING confidence: 0.7 (high), MCL clustering inflation parameter: 4. For functional analysis of each cluster, Enrichr[32] (https://maayanlab.cloud/Enrichr) and gene ontology (GO) enrichment analysis (http://geneontology.org) web server were utilized.

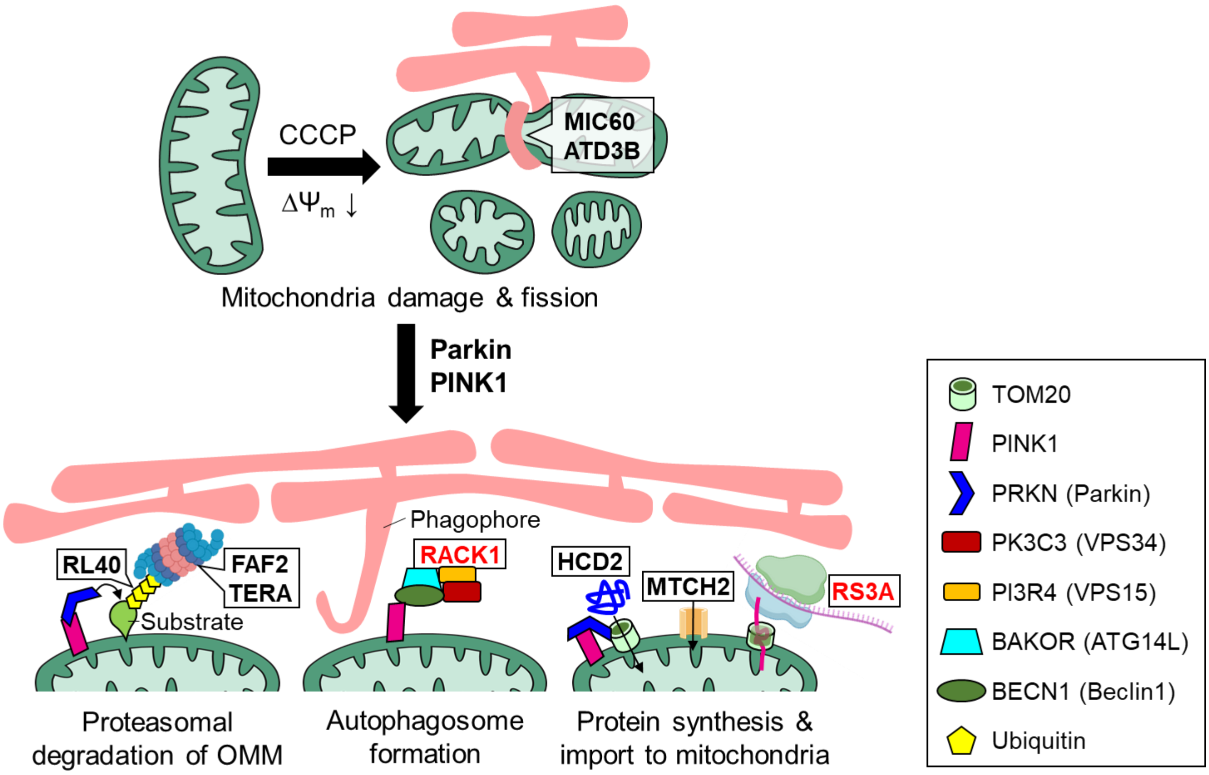

Supplementary Figure 15. Schematic description of roles of nine exclusively identified proteins from OrthoID in CCCP-treated cells at ER-mito junction. Protein names in red (RACK1 and RS3A) are depicted with suggested roles in participating autophagosome formation and protein synthesis during mitophagy, respectively, based on their known functions described in Table S3.

Supplementary Table 4. Known functions of exclusively identified proteins from OrthoID in CCCP-treated cells.

**Accession**	**Gene name**	**Localization (GOCC*)**	**Known function**
Q96CS3	FAF2	ERM	- Proteasomal degradation / ER-associated degradation (ERAD)[33]<br>- Regulation of ER-mito contacts by alteration of membrane composition[34]
P55072	TERA	ERM	- Proteasomal degradation / ER-associated degradation (ERAD)[33]
P61247	RS3A	ER	- Ribosome assembly, protein translation[35]<br>- Regulating mitochondrial function[36]
P62987	RL40	ERM, OMM	- Ubiquitination, a regulation of the ribosomal protein complex[37]
Q99714	HCD2	Mito matrix	- Mitigation of CCCP-induced mito degradation, Increase import from cytosol to mitochondria through TOM20[38]
Q9Y6C9	MTCH2	OMM	- Tail-anchored mitochondrial protein insertase[39]
P63244	RACK1	Mitochondrion	- Participate in the formation of autophagosome and induction of autophagy[40]
Q16891	MIC60	IMM	- MICOS complex (interaction with SAM and TOM complex at OMM and forms OMM-IMM contact site crucial for mitochondrial dynamics)[41]<br>- IMM and cristae structure organization important for mtDNA synthesis where the mito-fission occurs[42-43]
Q5T9A4	ATD3B	IMM	- Regulation of IMM structure[44]

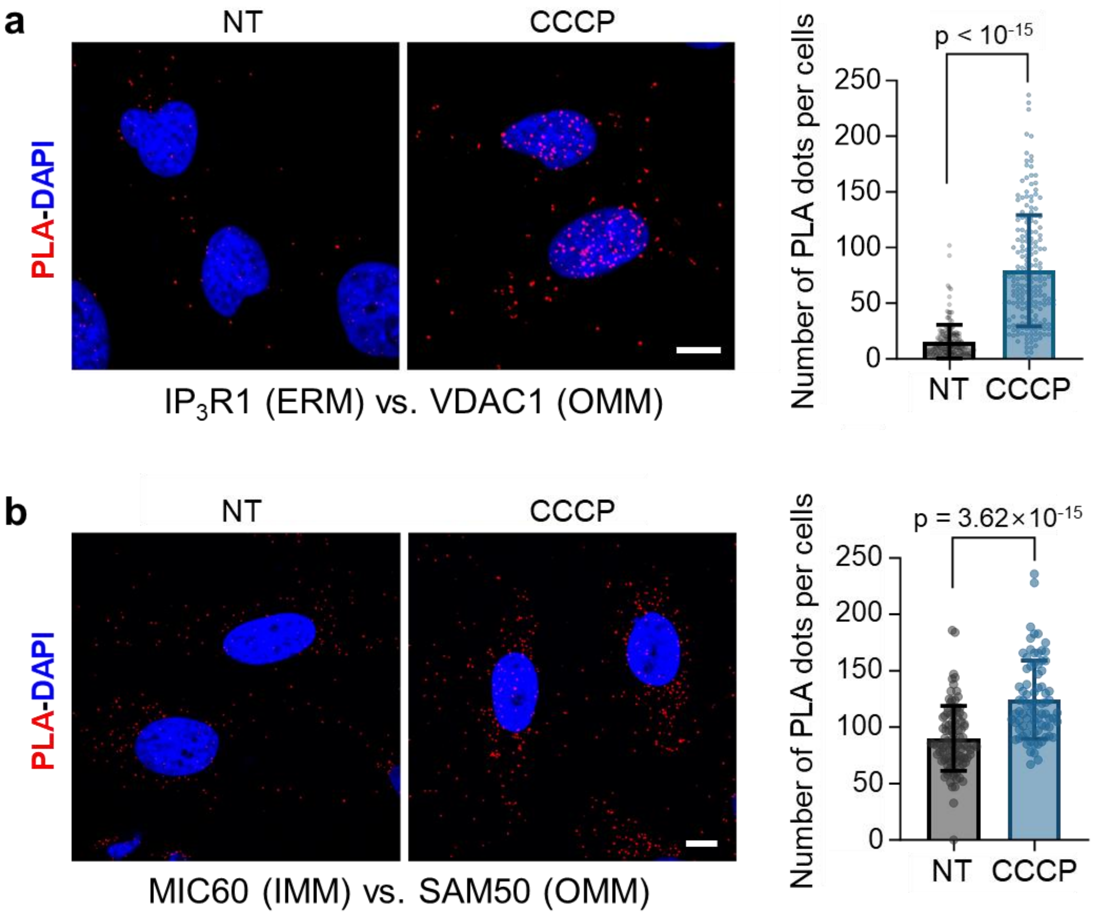

Supplementary Figure 16. Increased interactions between proteins at ERM, OMM and IMM detected by proximity ligation assay (PLA) under CLSM.

(a) Representative CLSM images and statistical analysis for counting the number of PLA dots in non-treated and CCCP-treated cells. Reaction performed between IP_3R1 as an ERM marker protein and VDAC1 as an OMM marker protein. (n = 180 cells for Non-treated and 194 cells for CCCP-treated samples were examined in three independent experiments.) Scale bar: 10 µm.

(b) Representative CLSM images and statistical analysis for counting the number of PLA dots in non-treated and CCCP-treated cells. Reaction performed between MIC60 as an IMM marker protein and SAM50 as an OMM marker protein. (n = 100 cells for Non-treated and 80 cells for CCCP-treated samples were examined in three independent experiments.) Scale bar: 10 µm. The error bar in panels a and b represents the mean ± SD and the numbers in the graph represent p-values. Two-tailed unpaired Student's t-test was used to assess statistical differences. Source data are provided as a Source Data file.

Supplemental References

1. Cho, I.-T.; Adelmant, G.; Lim, Y.; Marto, J. A.; Cho, G.; Golden, J. A., Ascorbate peroxidase proximity labeling coupled with biochemical fractionation identifies promoters of endoplasmic reticulum-mitochondrial contacts. *J. Biol. Chem.* **2017,** *292* (39), 16382-16392.
2. Hung, V.; Lam, S. S.; Udeshi, N. D.; Svinkina, T.; Guzman, G.; Mootha, V. K.; Carr, S. A.; Ting, A. Y., Proteomic mapping of cytosol-facing outer mitochondrial and ER membranes in living human cells by proximity biotinylation. *eLife* **2017,** *6*, e24463.
3. Kwak, C.; Shin, S.; Park, J.-S.; Jung, M.; Nhung, T. T. M.; Kang, M.-G.; Lee, C.; Kwon, T.-H.; Park, S. K.; Mun, J. Y.; Kim, J.-S.; Rhee, H.-W., Contact-ID, a tool for profiling organelle contact sites, reveals regulatory proteins of mitochondrial-associated membrane formation. *Proceedings of the National Academy of Sciences* **2020,** *117* (22), 12109-12120.
4. Cho, K. F.; Branon, T. C.; Rajeev, S.; Svinkina, T.; Udeshi, N. D.; Thoudam, T.; Kwak, C.; Rhee, H.-W.; Lee, I.-K.; Carr, S. A.; Ting, A. Y., Split-TurboID enables contact-dependent proximity labeling in cells. *Proceedings of the National Academy of Sciences* **2020,** *117* (22), 12143-12154.
5. Doghman-Bouguerra, M.; Lalli, E., The ER-mitochondria couple: In life and death from steroidogenesis to tumorigenesis. *Molecular and Cellular Endocrinology* **2017,** *441*, 176-184.
6. Csordás, G.; Weaver, D.; Hajnóczky, G., Endoplasmic Reticulum–Mitochondrial Contactology: Structure and Signaling Functions. *Trends Cell Biol.* **2018,** *28* (7), 523-540.
7. Fujimoto, M.; Hayashi, T., New insights into the role of mitochondria-associated endoplasmic reticulum membrane. *International review of cell and molecular biology* **2011,** *292*, 73-117.
8. Yang, M.; Li, C.; Yang, S.; Xiao, Y.; Xiong, X.; Chen, W.; Zhao, H.; Zhang, Q.; Han, Y.; Sun, L., Mitochondria-Associated ER Membranes – The Origin Site of Autophagy. *Front. Cell Dev. Biol.* **2020,** *8*.
9. Vance, J. E., MAM (mitochondria-associated membranes) in mammalian cells: lipids and beyond. *Biochim. Biophys. Acta* **2014,** *1841* (4), 595-609.
10. Kumar, V.; Maity, S., ER Stress-Sensor Proteins and ER-Mitochondrial Crosstalk—Signaling Beyond (ER) Stress Response. *Biomolecules* **2021,** *11* (2), 173.
11. Chu, Q.; Martinez, T. F.; Novak, S. W.; Donaldson, C. J.; Tan, D.; Vaughan, J. M.; Chang, T.; Diedrich, J. K.; Andrade, L.; Kim, A.; Zhang, T.; Manor, U.; Saghatelian, A., Regulation of the ER stress response by a mitochondrial microprotein. *Nature Communications* **2019,** *10* (1), 4883.
12. Guardia-Laguarta, C.; Area-Gomez, E.; Rüb, C.; Liu, Y.; Magrané, J.; Becker, D.; Voos, W.; Schon, E. A.; Przedborski, S., α-Synuclein is localized to mitochondria-associated ER membranes. *The Journal of neuroscience : the official journal of the Society for Neuroscience* **2014,** *34* (1), 249-59.
13. Manganelli, V.; Matarrese, P.; Antonioli, M.; Gambardella, L.; Vescovo, T.; Gretzmeier, C.; Longo, A.; Capozzi, A.; Recalchi, S.; Riitano, G.; Misasi, R.; Dengjel, J.; Malorni, W.; Fimia, G. M.; Sorice, M.; Garofalo, T., Raft-like lipid microdomains drive autophagy initiation via AMBRA1-ERLIN1 molecular association within MAMs. *Autophagy* **2021,** *17* (9), 2528-2548.
14. Browman, D. T.; Resek, M. E.; Zajchowski, L. D.; Robbins, S. M., Erlin-1 and erlin-2 are novel members of the prohibitin family of proteins that define lipid-raft-like domains of the ER. *J. Cell Sci.* **2006,** *119* (Pt 15), 3149-60.
15. Gilady, S. Y.; Bui, M.; Lynes, E. M.; Benson, M. D.; Watts, R.; Vance, J. E.; Simmen, T., Ero1α requires oxidizing and normoxic conditions to localize to the mitochondria-associated membrane (MAM). *Cell Stress and Chaperones* **2010,** *15* (5), 619-629.
16. Li, Y.; Camacho, P., Ca^{2+}-dependent redox modulation of SERCA 2b by ERp57. *J. Cell Biol.* **2004,** *164* (1), 35-46.
17. He, J.; Shi, W.; Guo, Y.; Chai, Z., ERp57 modulates mitochondrial calcium uptake through the MCU. *Febs Lett.* **2014,** *588* (12), 2087-2094.
18. Harada, T.; Sada, R.; Osugi, Y.; Matsumoto, S.; Matsuda, T.; Hayashi-Nishino, M.; Nagai, T.; Harada, A.; Kikuchi, A., Palmitoylated CKAP4 regulates mitochondrial functions through an interaction with VDAC2 at ER–mitochondria contact sites. *J. Cell Sci.* **2020,** *133* (21).
19. Doghman-Bouguerra, M.; Granatiero, V.; Sbiera, S.; Sbiera, I.; Lacas-Gervais, S.; Brau, F.; Fassnacht, M.; Rizzuto, R.; Lalli, E., FATE1 antagonizes calcium- and drug-induced apoptosis by uncoupling ER and mitochondria. *EMBO reports* **2016,** *17* (9), 1264-1280.
20. Lahiri, S.; Chao, J. T.; Tavassoli, S.; Wong, A. K. O.; Choudhary, V.; Young, B. P.; Loewen, C. J. R.; Prinz, W. A., A Conserved Endoplasmic Reticulum Membrane Protein Complex (EMC) Facilitates Phospholipid Transfer from the ER to Mitochondria. *PLOS Biology* **2014,** *12* (10), e1001969.
21. Pitts, K. R.; Yoon, Y.; Krueger, E. W.; McNiven, M. A., The dynamin-like protein DLP1 is essential for normal

distribution and morphology of the endoplasmic reticulum and mitochondria in mammalian cells. *Mol. Biol. Cell.* **1999,** *10* (12), 4403-17.
22. Yoon, Y.; Pitts, K. R.; Dahan, S.; McNiven, M. A., A novel dynamin-like protein associates with cytoplasmic vesicles and tubules of the endoplasmic reticulum in mammalian cells. *J. Cell Biol.* **1998,** *140* (4), 779-93.
23. Friedman, J. R.; Lackner, L. L.; West, M.; DiBenedetto, J. R.; Nunnari, J.; Voeltz, G. K., ER Tubules Mark Sites of Mitochondrial Division. *Science* **2011,** *334* (6054), 358-362.
24. Iwasawa, R.; Mahul-Mellier, A. L.; Datler, C.; Pazarentzos, E.; Grimm, S., Fis1 and Bap31 bridge the mitochondria-ER interface to establish a platform for apoptosis induction. *The EMBO journal* **2011,** *30* (3), 556-68.
25. Chen, Y. F.; Kao, C. H.; Chen, Y. T.; Wang, C. H.; Wu, C. Y.; Tsai, C. Y.; Liu, F. C.; Yang, C. W.; Wei, Y. H.; Hsu, M. T.; Tsai, S. F.; Tsai, T. F., Cisd2 deficiency drives premature aging and causes mitochondria-mediated defects in mice. *Genes Dev.* **2009,** *23* (10), 1183-94.
26. Szabadkai , G. r.; Bianchi , K.; Várnai , P. t.; De Stefani , D.; Wieckowski , M. R.; Cavagna , D.; Nagy , A. I.; Balla , T. s.; Rizzuto , R., Chaperone-mediated coupling of endoplasmic reticulum and mitochondrial Ca^{2+} channels. *J. Cell Biol.* **2006,** *175* (6), 901-911.
27. Groisman, B.; Shenkman, M.; Ron, E.; Lederkremer, G. Z., Mannose trimming is required for delivery of a glycoprotein from EDEM1 to XTP3-B and to late endoplasmic reticulum-associated degradation steps. *J Biol Chem* **2011,** *286* (2), 1292-300.
28. Zhemkov, V.; Geva, M.; Hayden, M. R.; Bezprozvanny, I., Sigma-1 Receptor (S1R) Interaction with Cholesterol: Mechanisms of S1R Activation and Its Role in Neurodegenerative Diseases. *Int. J. Mol. Sci.* **2021,** *22* (8).
29. Simsek, D.; Tiu, G. C.; Flynn, R. A.; Byeon, G. W.; Leppek, K.; Xu, A. F.; Chang, H. Y.; Barna, M., The Mammalian Ribo-interactome Reveals Ribosome Functional Diversity and Heterogeneity. *Cell* **2017,** *169* (6), 1051-1065 e18.
30. Liu, F.; Ma, F.; Wang, Y.; Hao, L.; Zeng, H.; Jia, C.; Wang, Y.; Liu, P.; Ong, I. M.; Li, B.; Chen, G.; Jiang, J.; Gong, S.; Li, L.; Xu, W., PKM2 methylation by CARM1 activates aerobic glycolysis to promote tumorigenesis. *Nat. Cell Biol.* **2017,** *19* (11), 1358-1370.
31. Chen, J.; Liu, C. C.; Li, Q.; Nowak, C.; Bu, G.; Wang, J., Two structural and functional domains of MESD required for proper folding and trafficking of LRP5/6. *Structure* **2011,** *19* (3), 313-23.
32. Chen, E. Y.; Tan, C. M.; Kou, Y.; Duan, Q.; Wang, Z.; Meirelles, G. V.; Clark, N. R.; Ma'ayan, A., Enrichr: interactive and collaborative HTML5 gene list enrichment analysis tool. *BMC Bioinformatics* **2013,** *14*, 128.
33. Zheng, J.; Cao, Y.; Yang, J.; Jiang, H., UBXD8 mediates mitochondria-associated degradation to restrain apoptosis and mitophagy. *EMBO Rep.* **2022,** *23* (10), e54859.
34. Ganji, R.; Paulo, J. A.; Xi, Y.; Kline, I.; Zhu, J.; Clemen, C. S.; Weihl, C. C.; Purdy, J. G.; Gygi, S. P.; Raman, M., The p97-UBXD8 complex regulates ER-Mitochondria contact sites by altering membrane lipid saturation and composition. *Nat. Commun.* **2023,** *14* (1), 638.
35. Zhou, X.; Liao, W. J.; Liao, J. M.; Liao, P.; Lu, H., Ribosomal proteins: functions beyond the ribosome. *J Mol Cell Biol* **2015,** *7* (2), 92-104.
36. Tang, Y.; He, Y.; Li, C.; Mu, W.; Zou, Y.; Liu, C.; Qian, S.; Zhang, F.; Pan, J.; Wang, Y.; Huang, H.; Pan, D.; Yang, P.; Mei, J.; Zeng, R.; Tang, Q. Q., RPS3A positively regulates the mitochondrial function of human periaortic adipose tissue and is associated with coronary artery diseases. *Cell Discov* **2018,** *4*, 52.
37. Kobayashi, M.; Oshima, S.; Maeyashiki, C.; Nibe, Y.; Otsubo, K.; Matsuzawa, Y.; Nemoto, Y.; Nagaishi, T.; Okamoto, R.; Tsuchiya, K.; Nakamura, T.; Watanabe, M., The ubiquitin hybrid gene UBA52 regulates ubiquitination of ribosome and sustains embryonic development. *Sci. Rep.* **2016,** *6* (1), 36780.
38. Mouton-Liger, F.; Jacoupy, M.; Corvol, J.-C.; Corti, O., PINK1/Parkin-Dependent Mitochondrial Surveillance: From Pleiotropy to Parkinson's Disease. *Front. Mol. Neurosci.* **2017,** *10.*
39. Guna, A.; Stevens, T. A.; Inglis, A. J.; Replogle, J. M.; Esantsi, T. K.; Muthukumar, G.; Shaffer, K. C. L.; Wang, M. L.; Pogson, A. N.; Jones, J. J.; Lomenick, B.; Chou, T.-F.; Weissman, J. S.; Voorhees, R. M., MTCH2 is a mitochondrial outer membrane protein insertase. *Science* **2022,** *378* (6617), 317-322.
40. Zhao, Y.; Wang, Q.; Qiu, G.; Zhou, S.; Jing, Z.; Wang, J.; Wang, W.; Cao, J.; Han, K.; Cheng, Q.; Shen, B.; Chen, Y.; Zhang, W. J.; Ma, Y.; Zhang, J., RACK1 Promotes Autophagy by Enhancing the Atg14L-Beclin 1-Vps34-Vps15 Complex Formation upon Phosphorylation by AMPK. *Cell Rep.* **2015,** *13* (7), 1407-1417.
41. Rampelt, H.; Zerbes, R. M.; van der Laan, M.; Pfanner, N., Role of the mitochondrial contact site and cristae organizing system in membrane architecture and dynamics. *Biochimica et Biophysica Acta (BBA) - Molecular Cell Research* **2017,** *1864* (4), 737-746.
42. Hu, C.; Shu, L.; Huang, X.; Yu, J.; Li, l.; Gong, L.; Yang, M.; Wu, Z.; Gao, Z.; Zhao, Y.; Chen, L.; Song, Z.,

OPA1 and MICOS Regulate mitochondrial crista dynamics and formation. *Cell Death & Disease* **2020,** *11* (10), 940.

43. Lewis, S. C.; Uchiyama, L. F.; Nunnari, J., ER-mitochondria contacts couple mtDNA synthesis with mitochondrial division in human cells. *Science (New York, N.Y.)* **2016,** *353* (6296), aaf5549.
44. Arguello, T.; Peralta, S.; Antonicka, H.; Gaidosh, G.; Diaz, F.; Tu, Y.-T.; Garcia, S.; Shiekhattar, R.; Barrientos, A.; Moraes, C. T., ATAD3A has a scaffolding role regulating mitochondria inner membrane structure and protein assembly. *Cell Reports* **2021,** *37* (12), 110139.

Uncropped Scans of Blots in Supplementary Figures

Supplementary Figure 2b, c

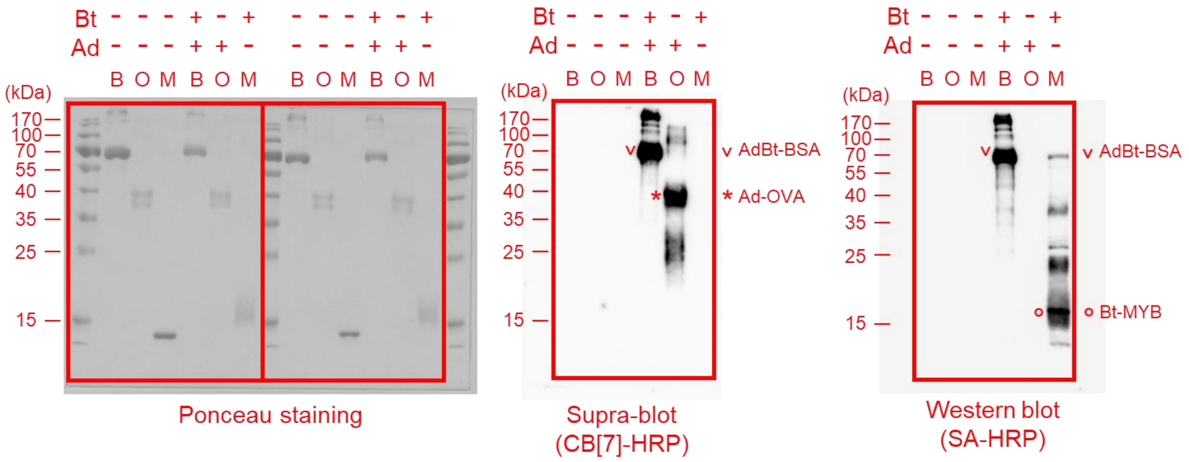

Supplementary Figure 5b

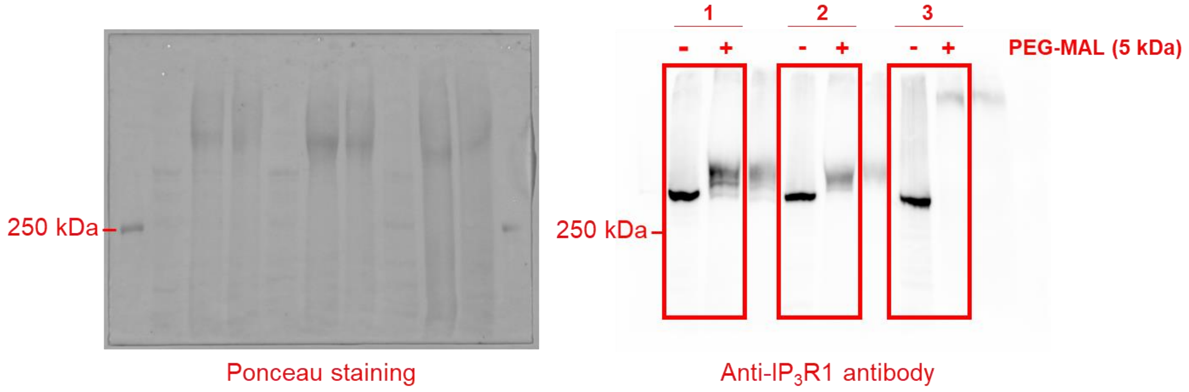

Supplementary Figure 7b

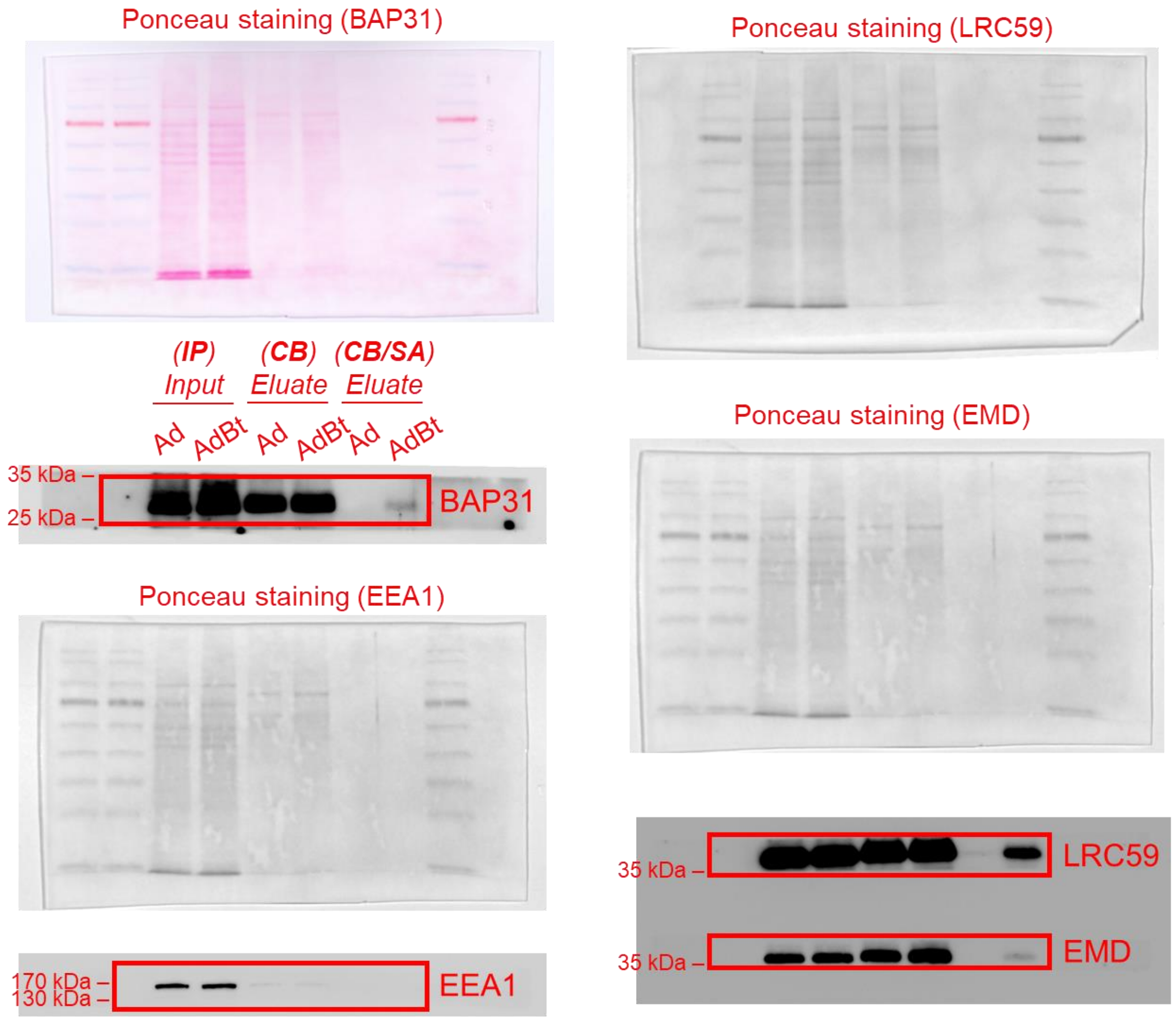

Supplementary Figure 9

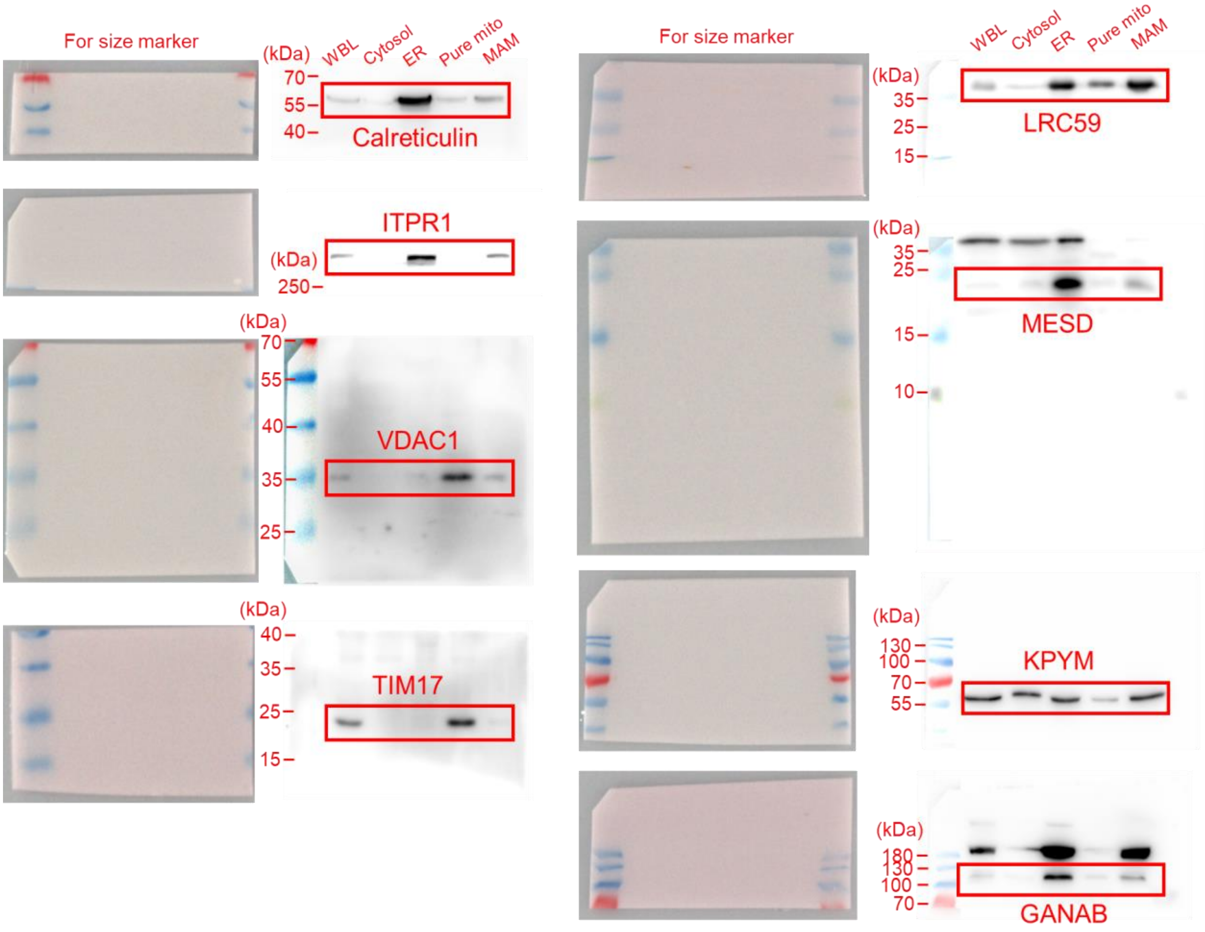

Supplementary Figure 11a

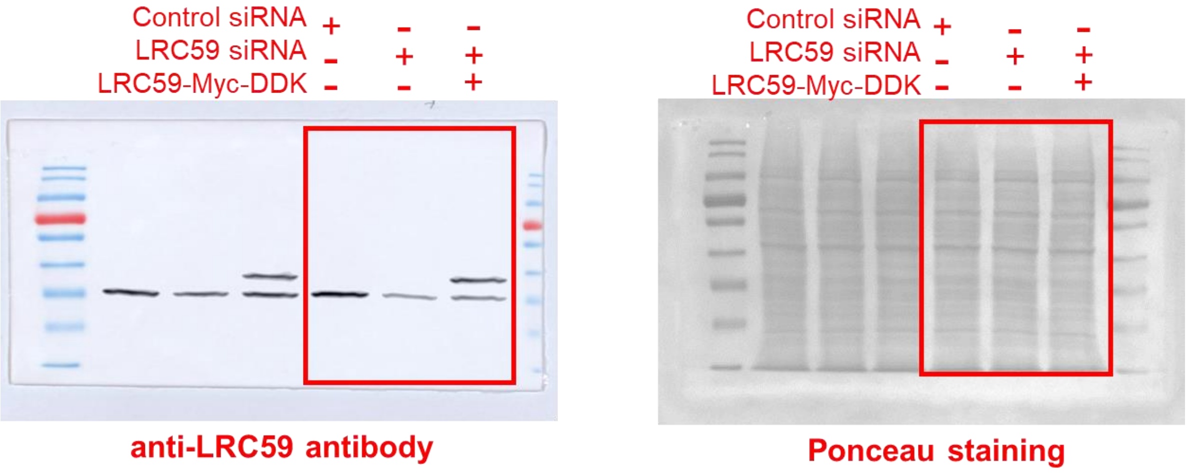

Supplement: Supplementary file 1 — Supplementary Information [file 41467_2024_46034_MOESM1_ESM.pdf]
