# Supplementary material for: OrthoID: profiling dynamic proteomes through time and space using mutually orthogonal chemical tools
Source: Nat Commun. 2024 Feb 29;15:1851. doi: 10.1038/s41467-024-46034-z (PMC10904832; doi:10.1038/s41467-024-46034-z)
Supplement: Supplementary file 3 — Description of Additional Supplementary Files [file 41467_2024_46034_MOESM3_ESM.pdf]

DESCRIPTION OF ADDITIONAL SUPPLEMENTARY FILES DOCUMENT

Supplementary Data 1: OrthoID results using model proteins

Supplementary Data 2: OrthoID results using stable cells

Supplementary Data 3: Topology analysis of the membrane proteins identified in OrthoID

Supplementary Data 4: OrthoID results using CCCP-treated stable cells

Supplementary Data 5: Comparison of identified PSMs in OrthoID between non-treated and CCCPtreated stable cells
