# Supplementary material for: OrthoID: profiling dynamic proteomes through time and space using mutually orthogonal chemical tools
Source: Nat Commun. 2024 Feb 29;15:1851. doi: 10.1038/s41467-024-46034-z (PMC10904832; doi:10.1038/s41467-024-46034-z)
Supplement: Supplementary file 9 — Reporting summary [file 41467_2024_46034_MOESM9_ESM.pdf]

nature portfolio

Corresponding author(s): Jong-Seo Kim, Hyun-Woo Rhee, Kyeng Min Park, Kimoon Kim

Last updated by author(s): Jan 31, 2024

Reporting Summary

Nature Portfolio wishes to improve the reproducibility of the work that we publish. This form provides structure for consistency and transparency in reporting. For further information on Nature Portfolio policies, see our Editorial Policies and the Editorial Policy Checklist.

Statistics

For all statistical analyses, confirm that the following items are present in the figure legend, table legend, main text, or Methods section.

n/a	Confirmed	
☐	☒	The exact sample size (*n*) for each experimental group/condition, given as a discrete number and unit of measurement
☐	☒	A statement on whether measurements were taken from distinct samples or whether the same sample was measured repeatedly
☐	☒	The statistical test(s) used AND whether they are one- or two-sided<br>*Only common tests should be described solely by name; describe more complex techniques in the Methods section.*
☒	☐	A description of all covariates tested
☐	☒	A description of any assumptions or corrections, such as tests of normality and adjustment for multiple comparisons
☐	☒	A full description of the statistical parameters including central tendency (e.g. means) or other basic estimates (e.g. regression coefficient) AND variation (e.g. standard deviation) or associated estimates of uncertainty (e.g. confidence intervals)
☐	☒	For null hypothesis testing, the test statistic (e.g. *F*, *t*, *r*) with confidence intervals, effect sizes, degrees of freedom and *P* value noted<br>*Give P values as exact values whenever suitable.*
☒	☐	For Bayesian analysis, information on the choice of priors and Markov chain Monte Carlo settings
☒	☐	For hierarchical and complex designs, identification of the appropriate level for tests and full reporting of outcomes
☐	☒	Estimates of effect sizes (e.g. Cohen's *d*, Pearson's *r*), indicating how they were calculated

Our web collection on statistics for biologists contains articles on many of the points above.

Software and code

Policy information about availability of computer code

Data collection	Xcalibur v4.1 (Thermo Fisher Scientific) was used for LC-MS/MS (proteomics) data collection. FV31S (Olympus) was used to obtain confocal microscope images. Tecnai G2 20 (Thermo Fisher Scientific) was used to obtain TEM images. Western and supra-blot data were collected with ImageQuant LAS500 (GE). Ascend 850 MHz spectrometer (Bruker) was used for taking 1H-NMR of Cy3-CB[7] and Ad. Autoflex Speed LRF MALDI-TOF instrument (Bruker) was used for MS analysis of Cy3-CB[7]. LTQ-XL mass spectrometer (Thermo Fisher Scientific) equipped with an electrospray ionization (ESI) source was used for MS analysis of Ad. Cary 660 FT-IR spectrometer (Agilent technologies) was used for CB[7]-bead characterization.
Data analysis	Proteome discoverer v2.3 (Thermo Fisher Scientific) was used to analyze LC-MS/MS data. GraphPad Prism 9.0 was used to perform statistical analysis. Cellsense 2.3 (Olympus) and Imaris 9.2 softwares were used to analyze confocal microscope images. Microscopy Image Browser (MIB) was used to analyze TEM images. ImageJ v1.53 was used for analysis of western blot band intensity. Topspin v3.7 (Bruker) was used to analyze 1H-NMR data.

For manuscripts utilizing custom algorithms or software that are central to the research but not yet described in published literature, software must be made available to editors and reviewers. We strongly encourage code deposition in a community repository (e.g. GitHub). See the Nature Portfolio guidelines for submitting code & software for further information.

Data

Policy information about availability of data

All manuscripts must include a data availability statement. This statement should provide the following information, where applicable:

- Accession codes, unique identifiers, or web links for publicly available datasets
- A description of any restrictions on data availability
- For clinical datasets or third party data, please ensure that the statement adheres to our policy

The MS data reported in this study have been deposited in the ProteomeXchange Consortium via the jPOST partner repository under accession code PXD040926 (https://proteomecentral.proteomexchange.org/cgi/GetDataset?ID=PXD040926) for ProteomeXchange and JPST002091 (https://repository.jpostdb.org/entry/JPST002091.1) for jPOST. The processed proteomics data are provided as Supplementary Data files. Source data are provided with this paper.

Research involving human participants, their data, or biological material

Policy information about studies with human participants or human data. See also policy information about sex, gender (identity/presentation), and sexual orientation and race, ethnicity and racism.

Reporting on sex and gender	N/A
Reporting on race, ethnicity, or other socially relevant groupings	N/A
Population characteristics	N/A
Recruitment	N/A
Ethics oversight	N/A

Note that full information on the approval of the study protocol must also be provided in the manuscript.

Field-specific reporting

Please select the one below that is the best fit for your research. If you are not sure, read the appropriate sections before making your selection.

☒ Life sciences ☐ Behavioural & social sciences ☐ Ecological, evolutionary & environmental sciences

For a reference copy of the document with all sections, see nature.com/documents/nr-reporting-summary-flat.pdf

Life sciences study design

All studies must disclose on these points even when the disclosure is negative.

Sample size	Sample sizes for all statistical analyses are given in the figure legends and in the Statistics and Reproducibility statement in the Methods section of the manuscript. No statistical method was used to predetermine sample size.
Data exclusions	No data were excluded.
Replication	Experiments were performed in three independent biological replicates, unless otherwise stated. All attempts at replication were successful.
Randomization	All samples were randomly allocated.
Blinding	We were blinded to group allocation.

Reporting for specific materials, systems and methods

We require information from authors about some types of materials, experimental systems and methods used in many studies. Here, indicate whether each material, system or method listed is relevant to your study. If you are not sure if a list item applies to your research, read the appropriate section before selecting a response.

Materials & experimental systems

n/a	Involved in the study
☐	☒ Antibodies
☐	☒ Eukaryotic cell lines
☒	☐ Palaeontology and archaeology
☐	☒ Animals and other organisms
☒	☐ Clinical data
☒	☐ Dual use research of concern
☒	☐ Plants

Methods

n/a	Involved in the study
☒	☐ ChIP-seq
☒	☐ Flow cytometry
☒	☐ MRI-based neuroimaging

Antibodies

Antibodies used	anti-BAP31 (Invitrogen, #MA3-002, CC-1, 1:200 dilution), anti-EMD (Proteintech, 10351-1-AP, 1:5000 dilution), anti-LRC59 (Proteintech, #27208-1-AP, 1:5000 dilution), anti-EEA1 (Cell Signaling Technology, #3288, C45B10, 1:500 dilution), anti-V5 (Invitrogen, #R960-25, SV5-Pk1, 1:1000 dilution), Alexa Fluor 488 conjugated Streptavidin (Invitrogen, #S11223, 1:500 dilution), anti-mouse IgG-HRP (Cell Signaling Technology, #7076, 1:1000 dilution), anti-rabbit IgG-HRP (Cell Signaling Technology, #7074, 1:1000 dilution), anti-VDAC1 (Proteintech, 55259-1-AP, 1:200 dilution), anti-IP3R1 (Santa Cruz, sc-271197, E-8, 1:200 dilution / Proteintech, 19962-1-AP, 1: 500 dilution), anti-SAM50 (Santa Cruz, sc-100493, SQ-7, 1:200 dilution), and anti-Mitofilin (Proteintech, 10179-1-AP, 1:200 dilution).
Validation	Validation of all commercially available primary antibodies can be found on the manufacturer's website or in the data sheet provided by the manufacturer, generally includes immunoblotting or immunohistochemical staining results.<br>1) anti-BAP31 (Invitrogen, #MA3-002), anti-V5 (Invitrogen, #R960-25): These antibodies were verified by knockdown or relative expression to ensure antibody binding to antigen by the manufacturer.<br>2) anti-EMD (Proteintech, 10351-1-AP), anti-LRC59 (Proteintech, #27208-1-AP), anti-VDAC1 (Proteintech, 55259-1-AP), anti-IP3R1 (Proteintech, 19962-1-AP), anti-Mitofilin (Proteintech, 10179-1-AP): Validation of these antibodies can be found on the manufacturer's website in the Validation Data Gallery, which includes immunoblotting or immunohistochemical staining results.<br>3) anti-EEA1 (Cell Signaling Technology, #3288), anti-mouse IgG-HRP (Cell Signaling Technology, #7076), anti-rabbit IgG-HRP (Cell Signaling Technology, #7074), anti-IP3R1 (Santa Cruz, sc-271197), anti-SAM50 (Santa Cruz, sc-100493): The validation of these antibodies can be found on each data sheet provided by the manufacturer on the website.

Eukaryotic cell lines

Policy information about cell lines and Sex and Gender in Research

Cell line source(s)	Flp-In™ T-REx™ 293 (Invitrogen, #R78007), HeLa (Korean Cell Line Bank, #10002) and HEK293T (ATCC, #CRL-3216)
Authentication	No authentication has been performed.
Mycoplasma contamination	All cell lines tested negative for mycoplasma contamination.
Commonly misidentified lines (See ICLAC register)	No commonly misidentified cell lines were used.

Animals and other research organisms

Policy information about studies involving animals; ARRIVE guidelines recommended for reporting animal research, and Sex and Gender in Research

Laboratory animals	12-week-old ICR (There is no need for housing as all the mice were sacrificed immediately after purchase.)
Wild animals	No wild animals were used.
Reporting on sex	The sex of the animal was not considered and all sexes were used for the experiment.
Field-collected samples	No field-collected samples were used.
Ethics oversight	All animal procedures were approved by the Institutional Animal Care and Use Committee (IACUC) of Pohang University of Science and Technology (POSTECH-2022-0085). All experiments were performed in accordance with the approved guidelines.

Note that full information on the approval of the study protocol must also be provided in the manuscript.

Plants

Seed stocks	*Report on the source of all seed stocks or other plant material used. If applicable, state the seed stock centre and catalogue number. If plant specimens were collected from the field, describe the collection location, date and sampling procedures.*
Novel plant genotypes	*Describe the methods by which all novel plant genotypes were produced. This includes those generated by transgenic approaches, gene editing, chemical/radiation-based mutagenesis and hybridization. For transgenic lines, describe the transformation method, the number of independent lines analyzed and the generation upon which experiments were performed. For gene-edited lines, describe the editor used, the endogenous sequence targeted for editing, the targeting guide RNA sequence (if applicable) and how the editor was applied.*
Authentication	*Describe any authentication procedures for each seed stock used or novel genotype generated. Describe any experiments used to assess the effect of a mutation and, where applicable, how potential secondary effects (e.g. second site T-DNA insertions, mosiacism, off-target gene editing) were examined.*
